# Echinococcoses and Tibetan Communities

**DOI:** 10.3201/eid1410.071636

**Published:** 2008-10

**Authors:** Philip S. Craig, Tiaoying Li, Jiamin Qiu, Ren Zhen, Qian Wang, Patrick Giraudoux, Akira Ito, David Heath, Bill Warnock, Peter Schantz, Wen Yang

**Affiliations:** University of Salford, Salford, UK (P.S. Craig); Sichuan Center for Disease Control and Prevention, Chengdu, People’s Republic of China (T. Li, J. Qui, Q. Wang, W. Yang); Aba Army Hospital, Markang, People’s Republic of China (R. Zhen); Universite de Franche-Comte, Besancon, France (P. Girardoux); Asahikawa Medical College, Asahikawa, Japan (A. Ito); Agresearch, Wallaceville Animal Research Centre, Upper Hutt, New Zealand (D. Heath); Boulder-Lhasa Sister City Project, Boulder, Colorado, USA (B. Warnock); Centers for Disease Control and Prevention, Atlanta, Georgia, USA (P. Schantz)

**Keywords:** hydatid, echinococcosis, Tibet, letter

**To the Editor:** The People’s Republic of China accounts for >500,000 cases of echinococcosis and more disability-associated life years (DALYs) lost because of this disease than any other world region (*1*,^2^). Hydatid cysts of *Echinococcus granulosus* (cystic echinococcosis [CE]), or the more pathogenic lesions with multiple vesicles caused by *E*. *multilocularis* infection (alveolar echinococcosis [AE]), usually grow slowly in the liver, so that severe illness and death may eventually occur in a high proportion of those with untreated infections (*3*,*4*). Apart from surgery, long-term anthelminthic therapy (>6 months) with the benzimidazole compound albendazole, although parasitostatic only, has a beneficial outcome in >50% of cases (*5*). To control the transmission of this zoonosis, veterinary public health measures must be emphasized (*6*).

In 2004 the Chinese Ministry of Health (MoH) undertook a nationwide assessment of 8 parasitic diseases, including malaria, schistosomiasis, and echinococcosis. To identify echinococcosis, 7 provincial MoHs carried out a mass abdominal screening of 34,500 persons using portable ultrasound scanners. The overall prevalence (2.5%) was highest in Tibetan communities in the Tibet Autonomous Region and in northwestern Sichuan and Qinghai Provinces (these latter regions form part of the eastern Tibetan Plateau). Collaborative studies involving the Sichuan Center for Disease Control and Prevention (based in Chengdu) and an international consortium of research institutes partly funded by the US National Institutes of Health (Bethesda, MD, USA) have shown an increasingly serious public health problem at the village, township, and county levels. In Shiqu County of Ganze Tibetan Autonomous Prefecture, 414 (12.9%) of nearly 3,199 persons surveyed by ultrasound (with serologic confirmation) exhibited CE or AE, including 19% in this category (*7*). The effects of human echinococcosis are substantial, with >50,000 DALYs lost in a population of 63,000 in Shiqu County (*8*).

Despite increased urbanization in China, >70% of Tibetans still live as seminomadic pastoralists on the high grasslands at an altitude >3,500 m. Most Tibetan herdsmen families keep at least 1 dog, and large numbers of ownerless stray dogs are tolerated by pastoralists and Buddhist monks. Risk factors for human echinococcosis (both CE and AE) in Tibetan communities usually include occupation, age (older persons are at higher risk), gender (higher risk for female), environment (pastoral landscapes), livestock ownership, and a history of dog ownership, as well as indicators of low socioeconomic status, including poor water quality and illiteracy (*7*,*9*) The prevalence levels of human AE in Ganze Tibetan Autonomous Prefecture (Sichuan Province) are among the highest recorded anywhere in the world. This situation presents a formidable challenge for early diagnosis, optimal affordable treatment, and prevention and control. Markham Hospital in Aba Tibetan Autonomous Prefecture (Sichuan) performed 1,200 operations for echinococcosis from 1992 through 2005, 20% for AE disease. For remote, high-altitude, pastoral Tibetan communities, however, long-term albendazole therapy is the only realistic treatment option, but regular follow-up of patients is difficult in these poorly accessible communities.

To address the public health concerns and consider options for controlling hydatidosis/echinococcosis in the eastern Tibetan Plateau, an International Workshop on Treatment, Prevention and Control of Echinococcosis was held in Chengdu in May 2006 with support from the Sichuan Center for Disease Control and Prevention, MoH Beijing, the New Zealand International Aid and Development Agency [NZIAD]), Fogarty-National Institutes of Health (USA), Xinjiang Medical University, and the Boulder-Lhasa Sister City Project. Recommendations stressed the following public health needs: improved treatment centers within the known disease-endemic counties or prefectures for long-term follow-up of patients after surgery and chemotherapy for both CE and AE disease, a better understanding of the epidemiology and ecology of transmission, and planning for pilot control interventions against both CE and AE transmission. NZAID made a detailed report of the implementation and effects of a pilot echinococcosis control program (2000–2006) in Datangma County, Ganze Tibetan Autonomous Prefecture (Sichuan Province). Problems occurred chiefly because of poor intersectoral cooperation, difficult logistics, cultural antagonism, lack of participatory planning, difficult access, treatment of dogs (with praziquantel), vaccination of livestock with the new EG95 vaccine (*6*), and lack of adequate surveillance of dog and livestock infection levels. The report indicated how many of these difficulties could be overcome. Consequently, the People’s Republic of China MoH and provincial disease control networks approved funding in July 2006 to initiate pilot intervention programs against echinococcosis in 17 Tibetan autonomous counties of northwest Sichuan. Control options initially focused on regular supervised dosing of owned dogs and stray dogs (with praziquantel) by local operatives from district disease control centers and on improving health education at primary healthcare levels. Surveillance relies on measuring regularly the degree of *Echinococcus* infection in dogs by using a coproantigen test. Also, a specific age cohort of schoolchildren is monitored by ultrasound and serologic testing each year to determine changes in the prevalence of the 2 diseases. Albendazole is provided free, and the cost of surgery for hydatid disease is also subsidized through the new National Rural Cooperative Medical Insurance System.

In addition to the major public health problem now being recognized for echinococcosis in Tibetan communities, their general health indices are low (higher prevalence of tuberculosis, bone diseases such as arthritis, and poorer health in general) because of living and working at altitudes >4,000 m, compared with those in most other areas of China. Access and outreach should be improved (in conjunction with animal health initiatives) (*10*) for effective delivery of treatment, vaccination, and health education packages to these largely scattered and marginalized pastoral communities.
